# Fatigue Design of Tubular Carbon–Aluminium Bonded Joints Under Constant- and Variable-Amplitude Fatigue

**DOI:** 10.3390/ma19040781

**Published:** 2026-02-17

**Authors:** Mauro Ricotta, Gianmaria Bettio, Giovanni Meneghetti

**Affiliations:** Department of Industrial Engineering, University of Padova, Via Venezia 1, 35131 Padova, Italy; mauro.ricotta@unipd.it (M.R.);

**Keywords:** tubular bonded joints, fatigue, variable-amplitude fatigue, Generalised Stress Intensity Factor

## Abstract

This study investigates the fatigue behaviour of carbon fibre–aluminium adhesively bonded tubular joints, representative of the suspension arm of a Formula SAE racing car, under both constant- and variable-amplitude fatigue loading. A linear elastic stress analysis was conducted using two-dimensional axisymmetric finite element models to determine the singular stress field parameters—specifically the Generalised Stress Intensity Factor (H_0_) and the stress singularity exponent (s)—at critical adhesive–adherend interfaces. Experimental tests under quasi-static loading and constant amplitude, as well as variable-amplitude fatigue conditions, were performed. The constant-amplitude fatigue data were reanalysed in terms of both nominal maximum shear stress and H_0_. The results show that the scatter index of the fatigue data was reduced by a factor of 1.46 when H_0_ was used as the fatigue-driving parameter, indicating an improved correlation of the experimental results. Variable-amplitude fatigue tests were interpreted using Miner’s cumulative damage rule, confirming the suitability of H_0_-based life estimation models even under realistic, variable-amplitude loading conditions. The results demonstrate that H_0_ is an effective parameter for rationalising fatigue performance of tubular bonded joints and highlight its potential for fatigue design in composite–metal structural applications.

## 1. Introduction

Adhesively bonded joints have emerged as an effective and increasingly adopted solution in the engineering of lightweight structures, particularly where traditional joining methods—such as welding, riveting, or bolting—are limited by geometric and/or material constraints. Among these, adhesively bonded tubular joints present an interesting combination of structural efficiency, ease of assembly, and potential for multimaterial integration. These characteristics make them particularly attractive for demanding applications in aerospace, automotive, wind energy, and civil infrastructure systems, where both static strength and fatigue durability are essential [1,2].

The growing interest in bonded tubular connections is largely driven by the need to design structures that are not only strong and lightweight, but also resistant to long-term cyclic loading [3,4]. Unlike single- or double-lap joints, tubular geometries introduce a more complex stress distribution, which complicates the estimations of fatigue behaviour. Stress concentrations near the joint ends, peel stresses induced by eccentricities or bending, and the three-dimensional nature of the stress state in tubular interfaces all contribute to challenges in both modelling and experimental evaluation. A comprehensive review of the theoretical models developed to evaluate stress distributions in axially loaded tubular joints was presented by Dragoni and Goglio [5]. In the present work, only selected features of these models will be briefly recalled, with attention limited to those most relevant to the current analysis. The foundational contribution by Lubkin and Reissner [6] represents one of the earliest analytical approaches to modelling tubular joints, based on assumptions adopted for flat lap joints [7]. Their model considers the adherends to be subjected to axial, shear, and bending loads, while the adhesive layer is assumed to transmit only shear and peel stresses, which vary along the axial direction. All other stress components are neglected. Subsequent studies proposed various modelling strategies, either simplifying or extending this original framework [8,9,10,11,12,13,14,15]. A number of simplified models treat the adherends as subjected solely to axial tension [9,10,11,12,13,14]. In contrast, more advanced approaches incorporate complex stress states within the adhesive, allowing for through-thickness stress variations [8,9,11,12,13,14] and/or the inclusion of additional stress components beyond shear and peel [8,10,11,12,13,14,16].

Understanding the stress distribution forms the basis for developing criteria to assess the static and/or fatigue strength of this type of joints. The approaches proposed in the literature can generally be classified into the following categories:

(a) Nominal stress-based approaches [17].
(b) Nominal strain-based approaches [18,19].
(c) Local energy-based approaches [3,20,21].
(d) Local cohesive Zone Model-based approaches [22].

The reader is referred to [23] for a comprehensive review of these methodologies.

In the present study, a local stress-based approach is employed for the fatigue life assessment of tubular carbon fibre–aluminium bonded joints. This approach has been successfully applied by the authors in previous works to rationalize the fatigue life to crack initiation in single-lap bonded joints subjected to constant-amplitude tension–tension fatigue loading [24,25,26]. The approach assumes that the number of cycles to crack initiation is governed by the intensity of the linear elastic singular stress field at the critical location in the joint, as originally proposed by Lefebvre and Dillard [27] and Lazzarin et al. [28] for bonded flat joints with isotropic adherends and adhesive.

The above-mentioned approaches have been extensively applied to assess the fatigue life of flat bonded joints, whereas only a limited number of papers are available in the literature concerning the tubular bonded joints [29,30]. Reedy and Guess [29] evaluated the axial strength and fatigue resistance of thick-walled, adhesively bonded E-glass/epoxy-to-aluminium tubular lap joints under tensile and compressive loading. Most joints employed untapered aluminium adherends of 12.5 mm thickness, while four specimens incorporated adherends tapered to 1 mm at the inner end of the bond. Failure consistently initiated at this inner bond end through adhesive cracking along the interface. Tension–tension fatigue tests revealed substantial performance degradation, with tensile strength and fatigue resistance exceeding their compressive counterparts for untapered adherends. Analytical models—including an elastic–perfectly plastic adhesive representation and a linear elastic fracture mechanics approach—indicated that bond failure occurs in regions of peak stress and that compressive loading of untapered adherends generates tensile peel stresses, explaining the tensile–compressive strength difference. Fracture mechanics analysis further estimated mode I crack opening under compressive loading, consistent with experimental results. Limited testing suggests that tapering aluminium adherends can mitigate this difference. The influence of adherend geometry and material properties on fracture parameters was additionally examined, together with the applicability of linear elastic fracture mechanics to the tested joints. Nayeb-Hashemi et al. [9] analysed the shear stress distribution in steel tubular joints adhesively bonded with epoxy and subjected to axial and torsional loading using a shear-lag framework, assuming that the shear stresses are non-uniformly distributed along the adhesive layer. For axial loading, the adhesive layer was modelled as sustaining only shear stresses, while the adherends were assumed to carry purely axial forces; the formulation also incorporated the through-thickness variation of shear stress within the adhesive. The influence of an internal void on the peak shear stress was subsequently quantified. A dimensionless parameter, denoted θ_a_, was introduced, and results indicated that the stress distribution is governed not only by θ_a_, but also by the tubes’ cross-sectional geometry. For tubes with identical cross-sectional areas, the shear stress profile along the overlap region was nearly symmetric. In [9], it was pointed out that when θ_a_ ≥ 6.7, a centrally located annular void occupying at least half of the overlap length had negligible impact on both the maximum shear stress and the corresponding failure load. Under torsional loading, the adhesive was idealized as deforming exclusively in circumferential shear, with all other modes neglected, while the tubes were assumed to deform in axial shear. The analysis accounted for shear stress variation across the adhesive thickness and introduced a second dimensionless parameter, θ_t_, specific to torsion. Findings revealed that shear stress in the bonded region depends on θ_t_ as well as the polar moments of inertia (J_1_ and J_2_) of the adherends. The effects of annular voids of varying size were also assessed for different θ_t_ values. Failure envelopes for tubular joints under pure axial, pure torsional, and combined loading were established experimentally. Based on these observations, a predictive damage model for joints subjected to combined axial–torsional cyclic loading was subsequently formulated, demonstrating good agreement with experimental fatigue lives. Hosseinzade et al. [30] investigated the influence of overlap length on the static load capacity and fatigue life of composite tubular bonded joints subjected to torsional loading. Experimental tests were conducted on joints with overlap lengths of 12 mm, 20 mm, 30 mm, and 40 mm, supported by finite element simulations that accounted for the nonlinear behaviour of both adhesive and adherends. The results showed that static capacity increased with overlap length, rising from 80.8 Nm at 12 mm to 128.3 Nm at 40 mm; however, the effective overlap length was identified as 12 mm, beyond which further gains in load-carrying capacity were negligible. Fatigue life improved by 48% when overlap length increased from 12 mm to 20 mm, with longer joints exhibiting fatigue behaviour comparable to that of the adherends. Failure modes shifted from cohesive to adherend failure as the overlap length increased

Due to the relatively limited amount of research available on the fatigue behaviour of tubular bonded joints, this investigation explores the applicability of the Generalised Stress Intensity Factor-based approach to synthesise both their static and fatigue behaviour; to the best of the authors’ knowledge, this approach has not been previously reported in the literature for tubular joints. To this end, the fatigue behaviour of carbon fibre–aluminium tubular bonded joints was investigated under both constant- and variable-amplitude fatigue loading. As a first step, the static and fatigue data were reanalysed in terms of nominal shear stress, in accordance with the relevant international standards adopted for the adhesive characterisation and for the design of bonded joints. Subsequently, the same data were reinterpreted in terms of the Generalised Stress Intensity Factor H_0_, evaluated at the critical point of the joints. Finally, the variable-amplitude fatigue test data were synthesized using the classical Miner’s cumulative damage rule.

## 2. Theoretical Background

**Figure 1:**
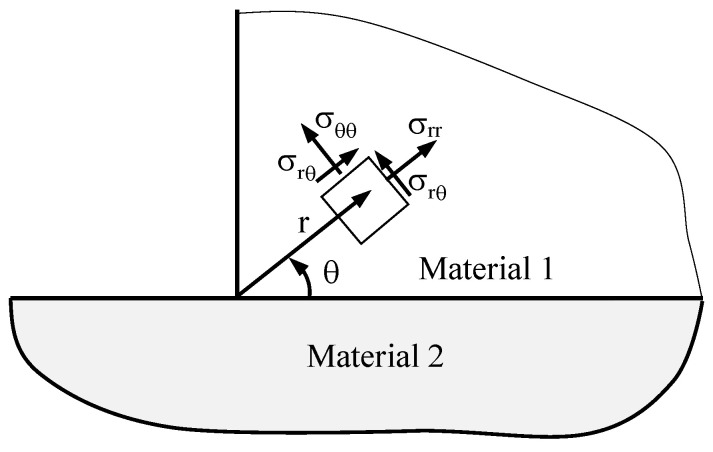
Schematic view of the zone close to the stress singularity point showing the polar reference system.

Linear elastic solutions for a bimaterial corner subjected to general loading conditions are available in the literature [31]. The singular stress fields near the interface corner can be described, in polar coordinates, as a sum of infinite terms plus a regular term, not depending on *r*

(1) $$\sigma_{ij}\left(r,\theta \right)=\sum_{k=0}^{\infty }T_{ij}^{k}\left(\theta \right)\cdot r^{\lambda_{k}}+\bar{T}\left(\theta \right)$$

where $T_{ij}^{k}\left(\theta \right)$ are angular functions dependent on polar angle θ, as well as on the loading conditions and geometry; *r* denotes the radial distance from the stress singularity point; and $\lambda_{k}$ are the eigenvalues of the problem. Following the approach proposed by Lazzarin et al. [28], the stress distributions close to the stress singularity point can be accurately approximated by a two-term stress expansion only, under the hypothesis that both first and second terms are in variable separable form. Each term can be represented as a radial component raised to the eigenvalue and an angular function. According to the frame of reference shown in Figure 1, the analytical formulation of the stress distributions can be rewritten as

(2) $$\sigma_{ij}\left(r,\theta \right)=H_{0}\cdot r^{s}\cdot f_{ij}^{\left(0\right)}\left(\theta \right)+H_{1}\cdot r^{t}\cdot f_{ij}^{\left(1\right)}\left(\theta \right)$$

where the eigenvalues s and t depend on corner geometry and elastic properties of materials, with the condition *s* < *t*. Furthermore, to maintain finite strain energy near the stress singularity, both eigenvalues cannot be lower than −1. The coefficients H_0_ and H_1_ represent the generalised SIFs associated with the first- and second-order terms, respectively, and do not correspond to mode I and mode II loading.

Close to the stress singularity points (i.e., as *r*→0), the leading term in the stress distribution predominates, and the stress field can be effectively approximated by the following expression:

(3) $$\sigma_{ij}\left(r,\theta \right)=H_{0}\cdot r^{s}\cdot f_{ij}^{\left(0\right)}\left(\theta \right)$$

The ability to characterize the stress field using solely the leading term of the asymptotic expansion is of particular significance, as it underpins the hypothesis that the fatigue performance of structural joints—specifically regarding crack initiation—is governed by the dominant term *H*_0_. This assumption, initially proposed in [28], was substantiated by experimental results presented in [24,26].

## 3. Specimen Preparation, Geometrical Details, and Test Procedure

**Figure 2:**
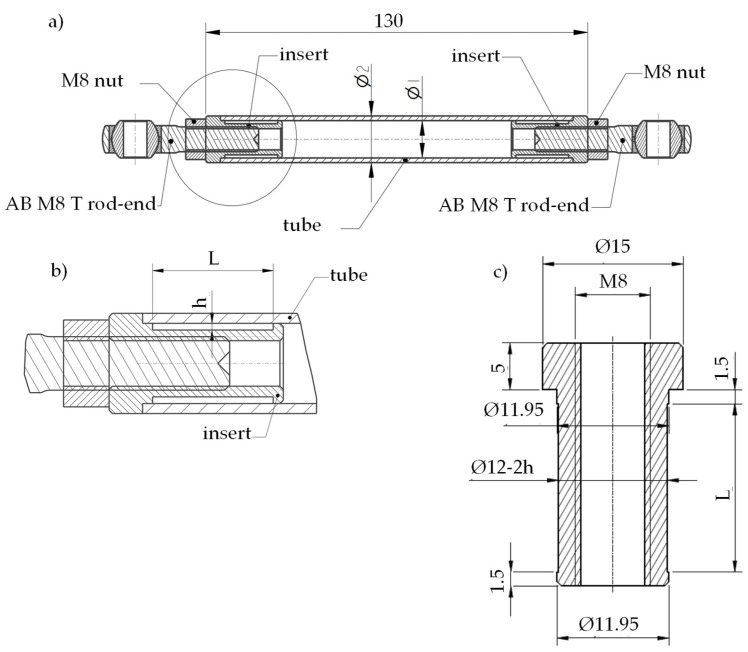
(**a**) Specimen’s geometry with detail of (**b**) bonded area and (**c**) 7075 aluminium insert.

Details of the tested specimens are shown in Figure 2a. They are representative of the front suspension triangle of a Formula SAE racing car developed at the University of Padova.

Carbon fibre-reinforced tubes with different layups were considered, as reported in the first column of Table 1, in detail, M46J unidirectional (UD) carbon fibre-reinforced epoxy matrix, GG 205 woven (W) carbon fibre–epoxy matrix. The influence of the overlap length L (namely 9 mm and 18 mm) and adhesive thickness h (namely 0.2 mm and 0.4 mm) were investigated as well (see Series 2 and 4 in Table 1). The rod ends were connected to the composite tubes using 7075 aluminium inserts, with the geometry shown in Figure 2b.

The internal surface of the tubes was sanded with 800-grit sandpaper, while the surfaces of the inserts were sandblasted. Then, the adherend surfaces were cleaned with nitro thinner. Finally, the composite tubes and the aluminium inserts were bonded using a brittle [25,32] two-component epoxy paste adhesive, EC-9323 B/A, provided by 3M (3M Italia, Pioltello, Italy). The adhesive thickness was guaranteed thanks to the geometry of the inserts. Specimens were tested after curing for at least 24 h at room temperature.

The elastic properties of the materials were assessed through quasi-static tensile testing of plain laminates manufactured from the same constituent materials used in tube production. Test specimens with off-axis angles of θ = 0°, 45°, and 90° were machined from a 500 mm × 500 mm plate and subsequently tested in accordance with ASTM D3039 standards [33]. For the characterisation of the elastic properties, a displacement rate of 2 mm/min was applied. For UD laminates with θ = 0° and 45°, as well as for woven laminates, the quasi-static tensile tests were conducted using a Schenck Hydropuls PSA 100 servo-hydraulic testing system (Instron, Milan, Italy) with a load capacity of 100 kN and a Trio Sistemi RT3 digital controller (Trio Sistemi, Bergamo, Italy). Conversely, due to the reduced mechanical strength of the θ = 90° UD laminates, an E05 StepLab electromechanical testing machine (StepLab, Resana, Italy) was employed, equipped with a 5 kN load cell and a STEP Lab Test Manager digital controller (StepLab, Resana, Italy). In all quasi-static tensile tests, axial strain was recorded using an MTS extensometer with a 25 mm gauge length (MTS, Turin, Italy).

In the case of bonded joints, quasi-static tensile tests were likewise performed under displacement control, adopting a displacement rate of 1 mm/min and were conducted using the aforementioned Schenck Hydropuls PSA 100 servo-hydraulic testing machine. The same testing system was also used for tension–tension fatigue tests (load ratio R = 0.05), under both constant- and variable-amplitude loading conditions. Fatigue test frequencies ranged from 1 Hz to 30 Hz, depending on the applied maximum force: the higher the applied force, the lower the applied load test frequency.

After the quasi-static and fatigue tests, the fracture surfaces were observed using a Dino-Lite AM4115ZT digital microscope (Italeco, Rivoli, Italy) and a Keyence VHX X1 optical-digital microscope (Keyence Italia, Milan, Italy).

The elastic properties of UD and woven laminae, as well as epoxy adhesive and aluminium insert, are listed in Table 2. The subscripts refer to the material reference coordinate system, where directions 1 and 2 define the lamina plane and direction 3 corresponds to the through-thickness direction, orthogonal to the lamina plane, as widely adopted in the literature (see [34,35] as examples).

## 4. Finite Element Models

**Figure 3:**
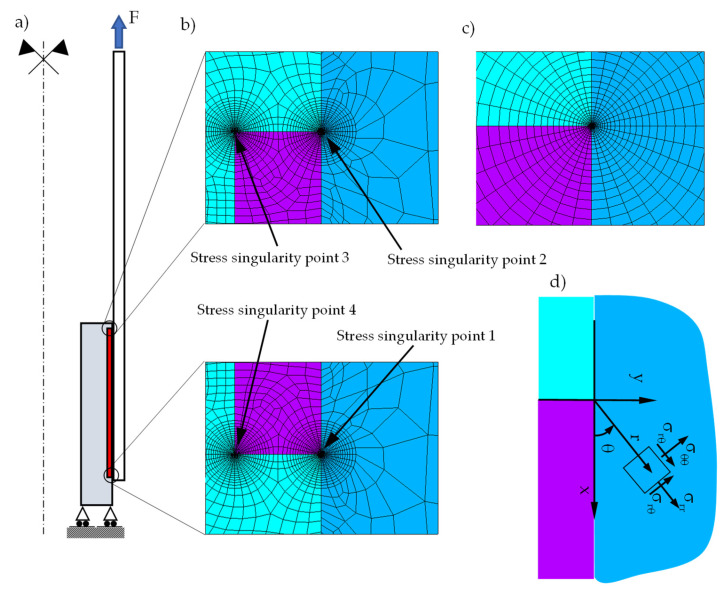
(**a**) Finite element model of tubular bonded joints with (**b**) numbering of the stress singularity points and (**c**) FE mesh detail close to the singular point with (**d**) an example of the local coordinate system adopted in stress singularity point 2.

To evaluate the Generalised Stress Intensity Factor H_0_ and the stress singularity exponent s (see Equation (3)), 2D axisymmetric linear elastic finite element (FE) models, representing the uncracked joint geometry, were developed using ANSYS 2020 R2 commercial software, employing eight-node PLANE183 elements. Regarding the material behaviour, the composite tube was modelled as an orthotropic material, while the adhesive layer and the aluminium insert were assumed to behave isotropically, using the material properties reported in Table 2. The boundary conditions and the applied load are sketched in Figure 3a. Figure 3b shows the four singularity points that characterise the FE model: 1 and 2 are located at the composite–adhesive–aluminium interface, whereas 3 and 4 are at the adhesive–aluminium interface. To accurately capture the asymptotic stress distribution near the singularity points, highly refined FE mesh patterns were employed. Following a mesh sensitivity analysis, the smallest element size was set equal to 1.7 × 10^−6^ mm close to the singularity, as reported in Figure 3c, in accordance with the meshing strategies reported in [25,26,28,36]. The element size was gradually increased up to 10^−1^ mm away from the singular regions.

## 5. Results of FE Analyses

**Figure 4:**
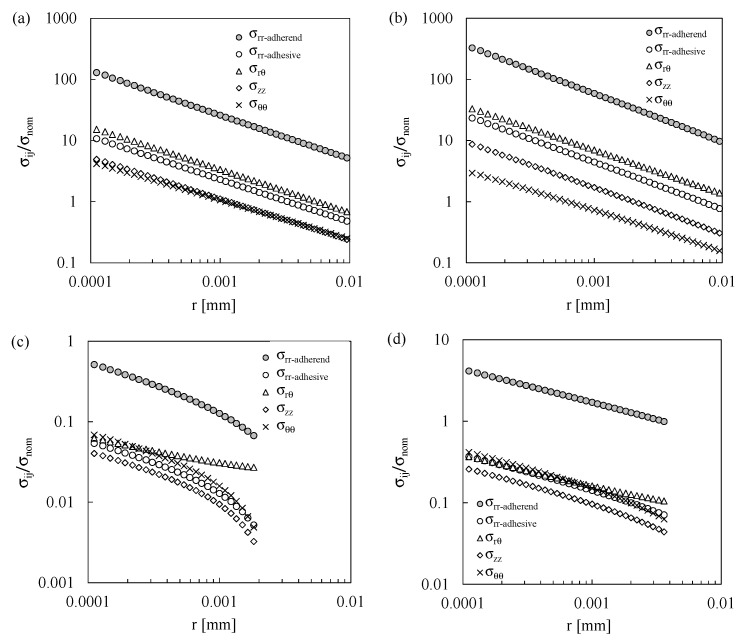
Normalised stress fields at θ = 0° for stress singularity point (**a**) 1, (**b**) 2, (**c**) 3, and (**d**) 4 of Series 1.

For the sake of brevity, only the linear elastic stress fields, normalised with respect to the nominal applied stress, σ_nom_, derived from FE analyses of Series 1, are presented in detail; more in detail Figure 4a–d correspond to stress singularities 1, 2, 3, and 4, respectively, with reference to the local coordinate system illustrated in Figure 3d. It is worth noting that, at stress singularity 3, with the exception of σ_rθ_, all remaining stress components exhibit negative values, particularly in the immediate vicinity of the stress singularity. In Figure 4c, absolute values are plotted to enable representation on a logarithmic scale. To comprehensively characterize the static and fatigue behaviour of these joints, and acknowledging that compressive normal stresses are generally less detrimental compared to tensile stresses, the compressive components were excluded from the evaluation of the H_0_ parameter, as discussed later.

The *H_0_* and *s* values were evaluated by fitting the nodal results relevant to the shear stress component, σ_rθ_, according to Equation (3) and assuming that the angular function f_rθ_ is equal to 1 at the adhesive–adherend interface (θ = 0).

**Figure 5:**
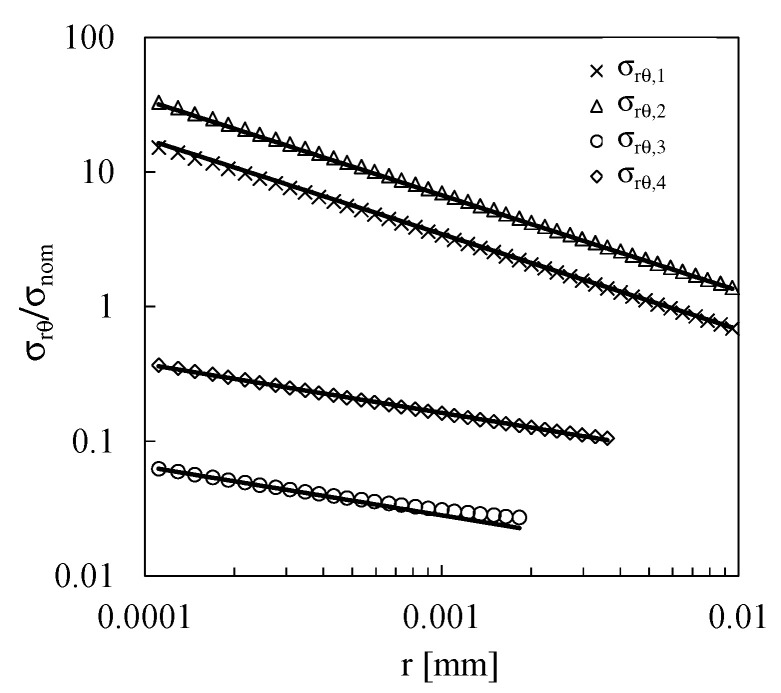
Normalised σ_rθ,_ stress fields (symbols) at θ = 0° for stress singularity points 1, 2, 3, and 4 fitted according to Equation (3) (solid lines).

The comparison between Equation (3) and the numerical results are plotted in Figure 5.

The complete results relevant to all series are listed in Table 3.

Table 3 reports both the H_0_ and the s values, showing that the highest absolute values of “*s*” correspond to the stress singularity points 1 and 2, where *s* = −0.709. Focusing on these points and considering the H_0_ values for all test series, the most critical point of stress singularity is point 2 (see [28] and the references reported therein). The effectiveness of the Generalised Stress Intensity Factor approach for fatigue assessment has already been reported in the literature for adhesively bonded joints, when epoxy-based adhesives are employed, for example, by Ishii et al. [37] and Lefebvre et al. [38]. A recent comprehensive review of this topic has been provided by Malekinejad et al. [2].

## 6. Results of Static and Fatigue Tests

The results of the quasi-static, constant-amplitude and variable-amplitude fatigue are presented in the next subsections. As a first step, the static and fatigue data are reanalysed in terms of the nominal shear stress in accordance with both the ASTM D1002 [39] and ASTM D3528 [40] standards adopted to characterize the static adhesive strength and the Eurocode 9 [41], which prescribes the fatigue design rules of adhesively bonded joints with aluminium adherends. Subsequently, static and fatigue data are reinterpreted according to the H_0_-based approach.

The use of the nominal shear stress is further supported by considering the stress distribution of these types of joints when subjected to purely axial external loads. As reported, for example, by Aimmanee et al. [42], indeed, the stress distribution is governed by the shear stress component, σ_rθ_, since all the normal stress components are generally much less significant. The only exception occurs at the overlap ends, which represent stress singularity regions, where the local stress state becomes singular and multiaxial and, therefore, can be captured using the H_0_-based approach.

### 6.1. Quasi-Static Results

**Figure 6:**
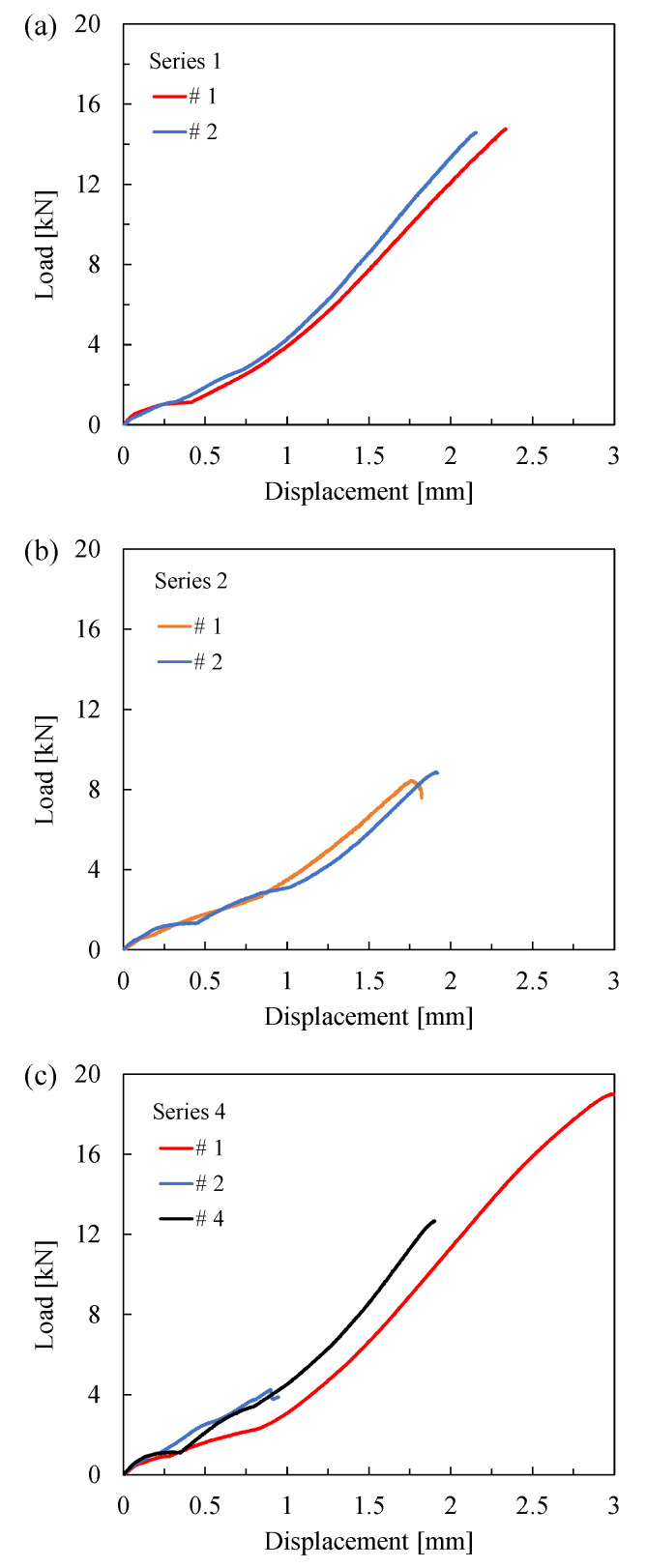
Load-displacement curves obtained for (**a**) Series 1, (**b**) Series 2, and (**c**) Series 4.

The load-displacement curves for the bonded joints are presented in Figure 6. Two tests were conducted for each series, with the exception of Series 4, for which three tests were performed due to the greater variability observed in this series.

As mentioned above, the results of the quasi-static tests were reanalysed in terms of the critical nominal shear stress, *τ*_nom,c_, as follows:

(4) $$\tau_{nom,c}=\frac{F_{c}}{\pi \cdot {Ø}_{1}\cdot L}$$

and in terms of H_0,c_ evaluated at the most critical stress singularity point (i.e., the number 2 from Table 3), and they are listed in Table 4. The average value, standard deviation, and coefficient of variation, defined as the ratio of the standard deviation to the average value ratio, are also provided. It can be observed that the data exhibited slightly lower scatter when the experimental results were reanalysed in terms of *τ*_nom,c_.

Concerning the values reported in Table 4, it is worth noting that the nominal shear strength accounts for variations in the overlap length, but it does not account for differences in adhesive thickness or in the carbon tube layup. As a consequence, when nominal shear strength is used, the resulting data cannot be regarded as statistically significant since they do not represent a single homogeneous population. Nevertheless, in practical applications, the use of nominal shear strength is widespread, as it represents the only practical means of comparison with the adhesive structural properties typically provided by manufacturers, according to ASTM D1002 [39] and ASTM D3528 [40] standards. Conversely, the Generalised Stress Intensity Factor H_0_ inherently accounts for all these parameters, thereby allowing the resulting mean values and scatter measures to be interpreted as statistically meaningful.

### 6.2. Constant-Amplitude Fatigue Test Results

**Figure 7:**
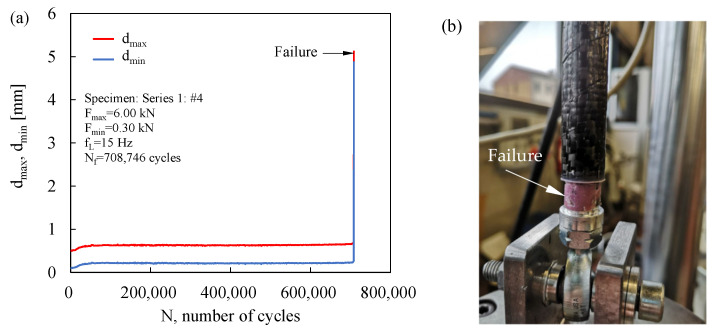
Example of (**a**) maximum and minimum displacement trends recorded during fatigue test and (**b**) detail of the bonding failure.

**Figure 8:**
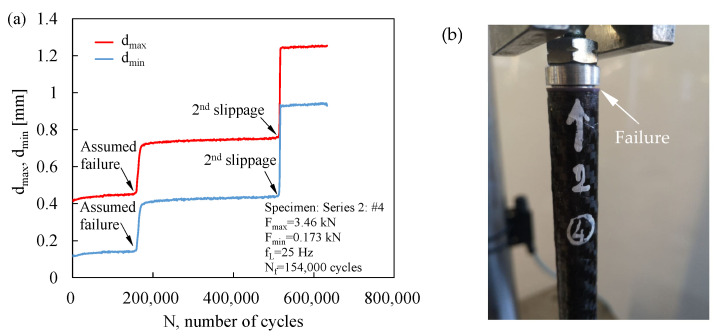
Example of (**a**) maximum and minimum displacement trends recorded during fatigue test and (**b**) detail of the bonding failure.

During the fatigue tests, the maximum, d_max_, and minimum, d_min_, displacements of the fatigue testing machine were monitored. Representative trends are shown in Figure 7 and Figure 8. Figure 7a illustrates that the displacement signals increased during the initial stage of fatigue life and subsequently stabilised; a sudden rise was associated with the final failure of the joint, as confirmed by the corresponding post-failure photograph shown in Figure 7b. In contrast, Figure 8a shows a stepwise evolution of the monitored signals. Visual inspection during testing revealed that the first displacement step coincided with the onset of insert pullout from the tube, as detailed in Figure 8b. Accordingly, in these cases, the number of cycles to failure was assumed to correspond to the number of cycles at which the initial pullout of the insert from the tube occurred. It is worth noting that no correlation was observed between the failure modes and either the applied load level or the number of cycles to joint failure.

Similarly to the quasi-static test results, the results of the fatigue tests were reanalysed in terms of the nominal maximum shear stress, as follows:

(5) $$\tau_{nom,max}=\frac{F_{max}}{\pi \cdot {Ø}_{1}\cdot L}$$

F_max_ is the maximum force imposed during the fatigue cycle.

**Figure 9:**
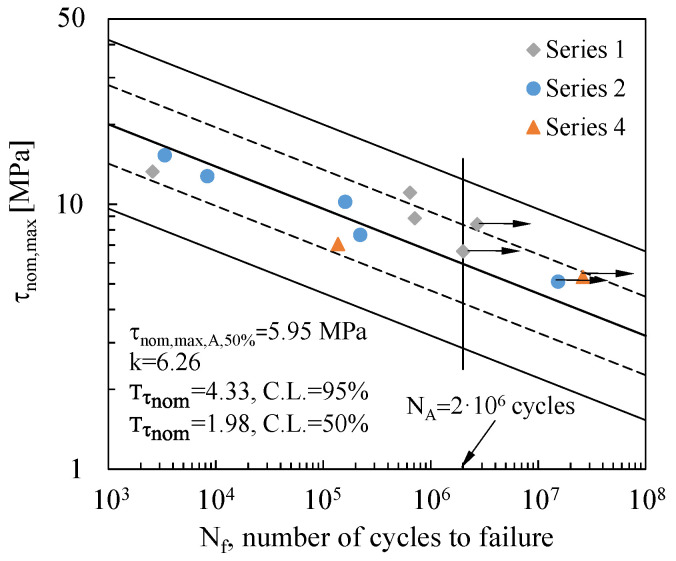
Fatigue data analysed in terms of nominal maximum shear stress, τ_nom,max_. Scatter bands are defined for 10 and 90% survival probabilities, with a confidence level of 95% (continuous lines) and 50% (dashed lines).

Fatigue data were statistically reanalysed according to ISO 12107–2012 standard [43]. The results are plotted in Figure 9, with the mean and the 10–90% survival probability curves fitting the experimental results with a confidence level (C.L.) of 95% (solid lines) and C.L. of 50% (dashed lines) according to Equation (6):

(6) $${\tau_{nom,max}}^{k}\cdot N_{f}=const$$

Figure 9 reports the inverse slope k of the curves, the $\tau_{nom,max,A,50\%}$ value at the reference fatigue life of N_A_ = 2 million cycles, and the $T_{\tau_{nom}}$ scatter indexes with C.L. of 95% and C.L. of 50%, defined as $\tau_{nom,max,10\%}/\tau_{nom,max,90\%}$. As expected, the statistical analysis would suggest increasing the size of the experimental dataset in order to raise the reliability of the curve to 90%.

**Figure 10:**
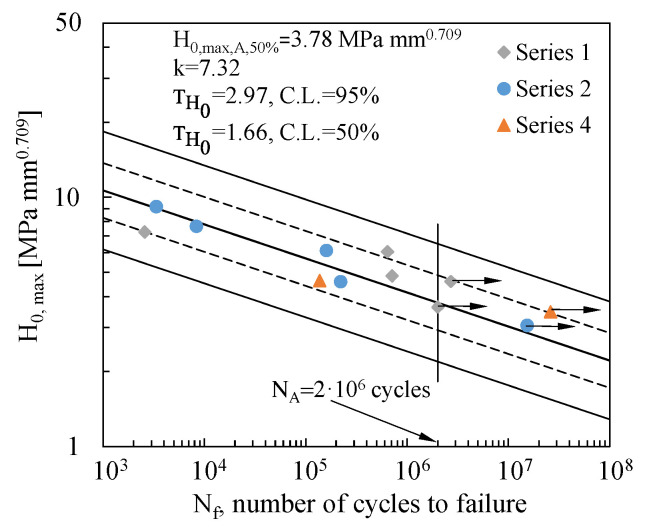
Fatigue data analysed in terms of the maximum value of the Generalised Stress Intensity Factor, H_0_,_max_. Scatter bands are defined for 10 and 90% survival probabilities, with a confidence level of 95% (continuous lines) and confidence level of 50% (dashed lines).

In this study, the number of cycles required for crack initiation could not be quantified due to the geometry of the bonded joints, which prevented direct visual inspection of the interfaces. For this reason, unlike previously performed investigations [24,26], the H_0_ parameter was used to reanalyse the fatigue data in terms of the number of cycles to failure, N_f_. According to the results reported in Table 3, the fatigue data were reanalysed, in accordance with [44], by using the H_0_, which was evaluated at the most critical stress singularity point, namely point 2. The results are plotted in Figure 10, with the mean and the 10–90% survival probability curves fitting the experimental data results with C.L. of 95% (solid lines) and C.L. of 50% (dashed lines), according to Equation (7):

(7) $${H_{0,max}}^{k}\cdot N_{f}=const$$

Figure 10 reports the inverse slope k of the curves, the mean $H_{0,max,A,50\%}$ value at the reference fatigue life of N_A_ = 2 million cycles, and the $T_{H_{0}}$ scatter indexes, defined as $H_{0,max,10\%}/H_{0,max,90\%}$. As expected, the statistical analysis would suggest increasing the size of the experimental dataset in order to raise the reliability of the curve to 90%.

Eventually, when comparing the relevant scatter indexes reported in Figure 9 and Figure 10, respectively, as expected, it can be noted that H_0_,_max_ provides a better correlation of the experimental data more effectively than $\tau_{nom,max}$. In light of this, H_0_,_max_ can be considered a promising parameter to synthesize the fatigue behaviour of tubular bonded joints.

### 6.3. Variable-Amplitude Fatigue Test Results

**Figure 11:**
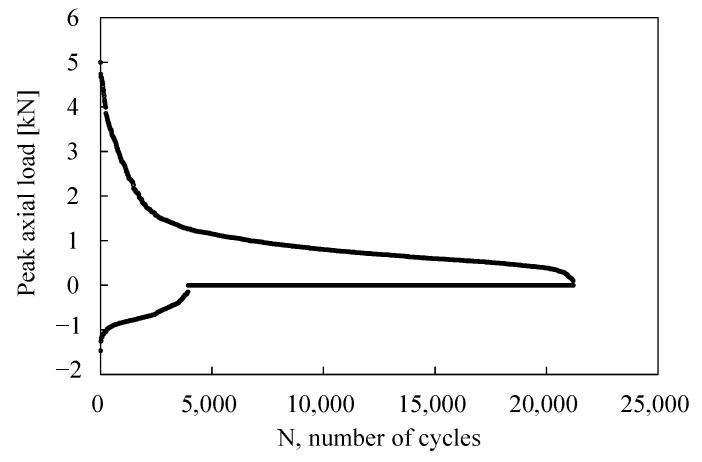
Load spectrum relevant to the lower arm of the front suspension triangle, for 36 km of racetrack.

As previously mentioned, the bonded joints analysed in the present study are representative of the front suspension triangle of a Formula SAE racing car developed at the University of Padova. In-field loads were previously measured on all suspension arms, revealing that the most heavily loaded component is the lower arm of the front suspension, which is predominantly subjected to tension–tension loading conditions. The load spectrum, originally representative of 3.6 km of racetrack, was extended by a factor of 10 in terms of number of cycles, corresponding to 36 km of racetrack, and is reported in Figure 11. As shown in this figure, the compressive component of the axial load is negligible compared with the tensile component. Consequently, the load spectrum was simplified as reported in Table 5, which lists the peak loads applied during the variable-amplitude fatigue (VAF) tests together with the corresponding number of cycles. The last column presents the peak force normalised with respect to that of the first block.

A conventional strategy for evaluating the fatigue response of materials under variable-amplitude fatigue (VAF) involves employing cumulative damage models. Over the years, numerous models have been introduced and extensively reviewed in the literature [23,44,45,46]. Among them, the Miner rule [47] remains one of the most frequently applied methods due to its ease of implementation. This approach quantifies damage through the following expression:

(8) $$D_{M}=\sum_{i=1}^{n_{b}}\frac{n_{i}}{N_{f_{i}}}$$

In this formulation, $D_{M}$ denotes the cumulative damage, n_i_ is the number of cycles in the i-th load block, N_fi_ represents the fatigue life under constant amplitude loading at the same load level, and n_b_ indicates the total number of applied blocks. According to this rule, structural failure is estimated when $D_{M}$ achieves unity. Despite its widespread use, this linear model presents notable shortcomings. Specifically, it does not account for the effects of load sequence interactions and inherently assumes a linear progression of damage. These limitations often lead to significant discrepancies between estimated and observed fatigue lives. Nonetheless, the Miner index remains widely adopted as a baseline metric for variable-amplitude fatigue damage. A cumulative damage value $D_{M}$ > 1 typically indicates the presence of retardation effects, whereas $D_{M}$ < 1 suggests that damage may have been accelerated during load sequence transitions.

In this study, the VAF tests were carried out by applying the load blocks reported in Table 5 until the specimen failed and by imposing a load ratio R = 0.05. Only in one case was the test interrupted, when the specimen reached 16,8 million cycles without failure.

**Figure 12:**
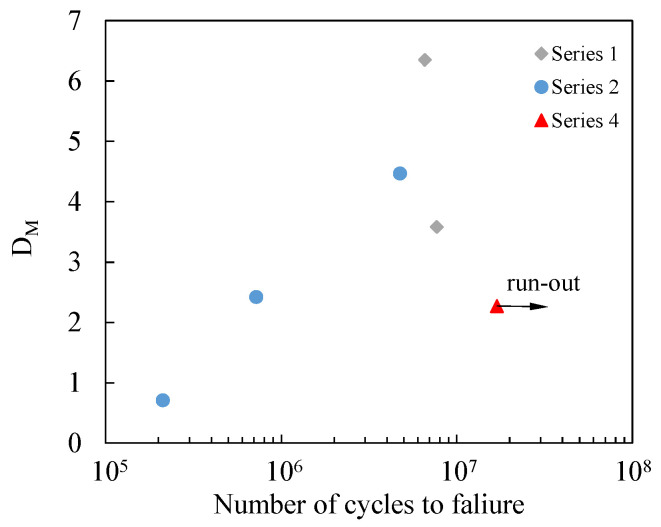
D_M_ values calculated during the variable-amplitude fatigue tests.

The effective D_M_ values calculated after the VAF tests are collected in Figure 12. It is evident that the D_M_ values are substantially greater than 1. These results are consistent with the findings reported by Sarfaraz et al. [48], who investigated the variable-amplitude fatigue behaviour of epoxy–adhesively bonded pultruded glass fibre-reinforced polyester resin. Their study examined the effects of loading sequence in two-block loading scenarios, as well as the influence of load-level transitions in multiblock loading conditions, considering different load ratios (R = 0.1 and R = 10). They observed that, under tension–tension loading conditions and high-to-low load transitions, the D_M_ values ranged from 1.72 to 6.17, whereas they were lower than 1 when low-to-high load transitions were applied. As opposed to these results, D_M_ values lower than 1 were reported by Erpolat et al. [49], who tested double-lap bonded joints, characterised by carbon fibre-reinforced AS4/8552 adherends bonded with Cytec 4535A, a single-part epoxy paste. In [49], double-lap joint specimens were subjected to both constant- and variable-amplitude fatigue loading regimes. Fatigue life estimations under variable-amplitude conditions were performed using Miner’s rule. The predictions markedly overestimated the actual fatigue life, suggesting a pronounced acceleration of crack propagation associated with load sequence effects inherent in the variable-amplitude spectrum. This acceleration was primarily attributed to fluctuations in mean stress levels; however, load overshoots were also identified as critical factors influencing crack nucleation.

**Figure 13:**
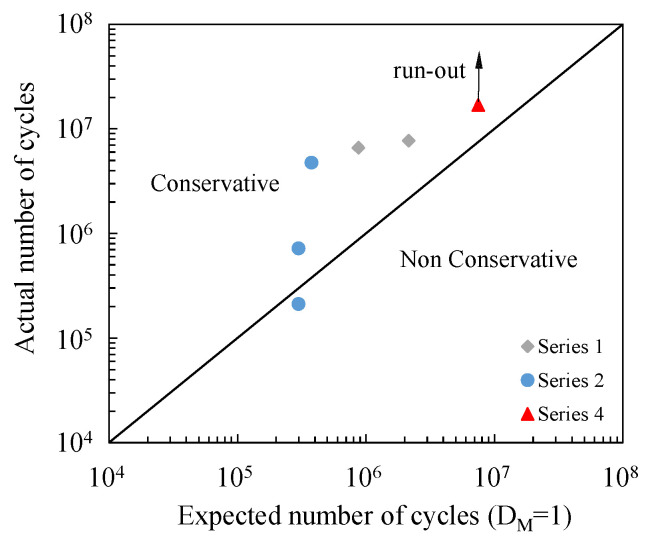
Actual number of cycles sustained by the specimens versus the predicted number of cycles to failure assuming D_M_ = 1. Predictions are conservative except for one case.

Figure 13 reports the actual number of cycles reached by the specimens plotted against the number of cycles to failure evaluated by imposing D_M_ = 1. It can be observed that, with the exception of a single case, the prediction is always conservative since the estimated number of cycles to failure is lower than the number of cycles actually sustained by the specimens.

## 7. Analysis of the Fracture Surfaces

After static and fatigue tests, the fracture surfaces were analysed to identify the damage mechanisms and, for the fatigue-tested specimens, the crack initiation site and the crack path, whenever possible.

**Figure 14:**
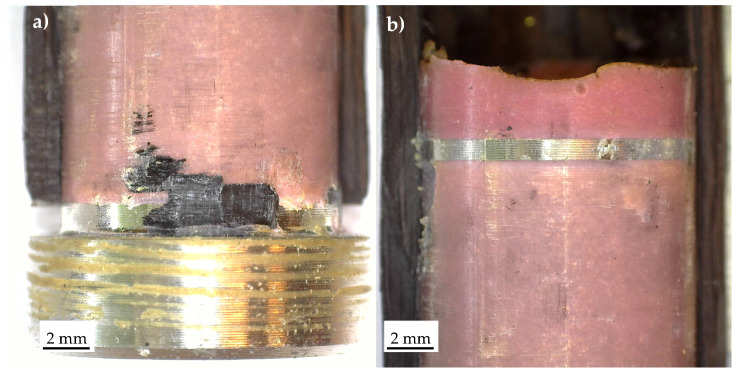
Characteristic fracture surface of a Series 1 specimen statically failed. Detail of stress singularity point (**a**) 1 and (**b**) 2, according to Figure 3.

Figure 14 presents the typical fracture surfaces observed in the case of Series 1-bonded joints after a quasi-static tensile test. The results indicate an adhesive failure at the composite–adhesive interface. Minor remnants of the carbon tube are visible in the stress singularity point 1, suggesting that only a very limited delamination occurred within the carbon tube.

**Figure 15:**
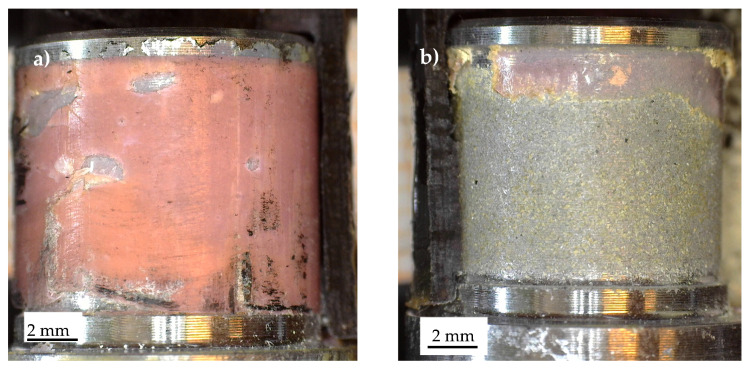
Characteristic fracture surface of Series 2 specimens statically failed. Detail of specimen number (**a**) 1 and (**b**) 2, according to Figure 3.

A more complex failure scenario was observed for Series 2. Two distinct fracture surfaces were identified, as shown in Figure 15. In specimen Series_2_1, failure occurred predominantly at the composite–adhesive interface, while in specimen Series_2_2, it occurred primarily at the aluminium–adhesive interface. In both cases, the failure mode was adhesive.

**Figure 16:**
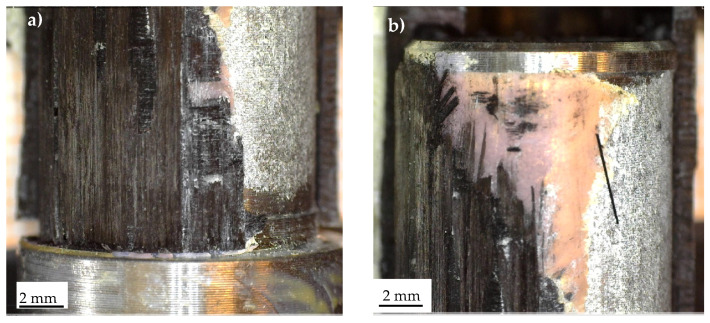
Characteristic fracture surface of a Series 4 specimen statically failed. Detail of stress singularity point (**a**) 1 and (**b**) 2, according to Figure 3.

A complex failure scenario was also observed in the Series 4 specimens, as shown in Figure 16, which highlights the stress singularity points 1 (Figure 16a) and 2 (Figure 16b). The fracture surfaces exhibit considerable complexity, with delamination in the carbon tube, particularly through the unidirectional (UD) plies, as well as adhesive failure along the composite–adhesive and aluminium–adhesive interfaces.

**Figure 17:**
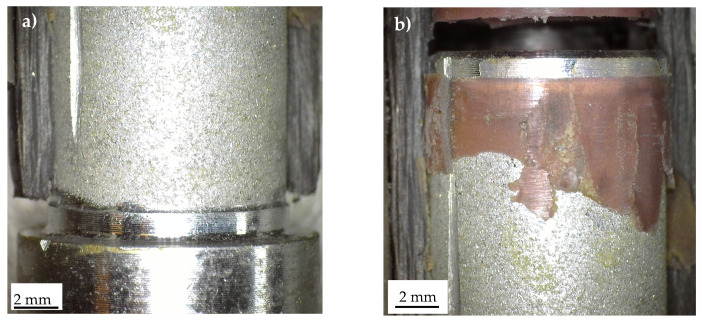
Characteristic fracture surface of a Series 1 specimen failed under constant-amplitude fatigue test (τ_nom,max_ = 11.05 MPa, N_f_ = 640,000 cycles). Detail of stress singularity point (**a**) 4 and (**b**) 2, according to Figure 3.

**Figure 18:**
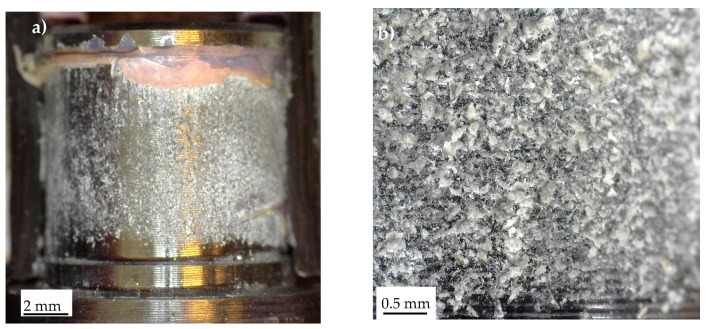
Characteristic fracture surface of a Series 2 specimen failed under constant-amplitude fatigue test (τ_nom,max_ = 12.75 MPa, N_f_ = 8319 cycles). Detail of bonded area (**a**) 1 and (**b**) of the stress singularity point 4, according to Figure 3.

**Figure 19:**
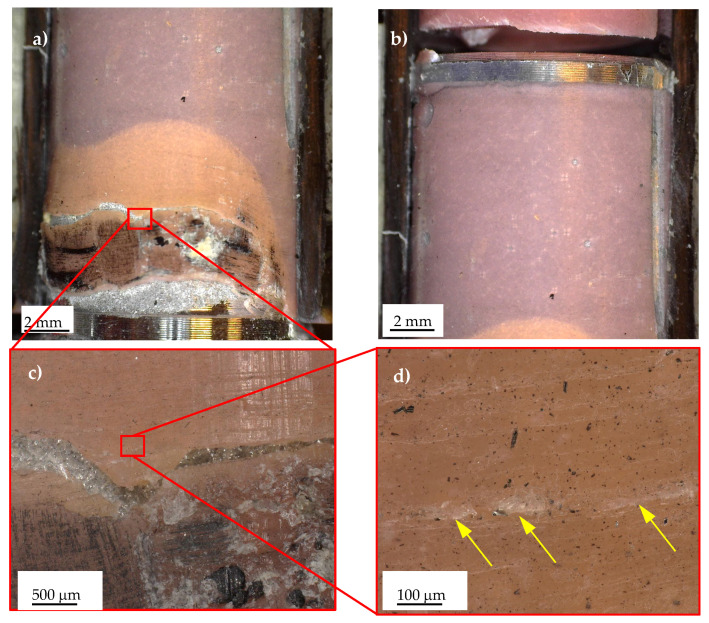
Characteristic fracture surface of a Series 4 specimen failed under constant-amplitude fatigue test. Detail of stress singularity point (**a**) 1 and (**b**) 2, according to Figure 3. (**c**,**d**) Adhesive plastic deformation. Figures (**a**,**b**) were obtained using Dino-Lite digital microscope, whereas (**c**,**d**) the Keyence digital microscope.

Regarding the constant-amplitude fatigue tests, representative fracture surfaces are presented in Figure 17, Figure 18 and Figure 19, corresponding to Series 1, 2, and 4, respectively. Figure 16a,b illustrates stress singularity points 4 and 2 of Series 1. It can be observed that the fatigue crack may have initiated from either stress singularity point 2 or stress singularity point 4. However, it is not possible to determine with certainty the exact location of crack initiation; in other words, the possibility that the crack originated from stress singularity point 2 cannot be excluded. A similar situation was observed for the Series 2 joints, where the fatigue crack may have initiated from either stress singularity point 2 or 4, as shown in Figure 18. In particular, Figure 18b highlights that the crack propagated predominantly along the aluminium–adhesive interface. This behaviour was consistently observed regardless of the applied stress amplitude.

Finally, Figure 19 illustrates the characteristic fracture surface observed for Series 4. In this case, it is reasonable to assume that the crack initiated at stress singularity point 2 and subsequently propagated toward point 1, where catastrophic failure likely occurred. The occurrence of catastrophic failure at point 1 may be reasonably inferred from the presence of multiple damage mechanisms in this region, including failure along the adhesive–aluminium interface and delamination of the carbon tube, the latter evidenced by fibre bundles remaining attached to the adhesive, as shown in Figure 19a. Moreover, plastic deformation of the adhesive, although limited, was observed, as highlighted in Figure 19d by the yellow arrows.

**Figure 20:**
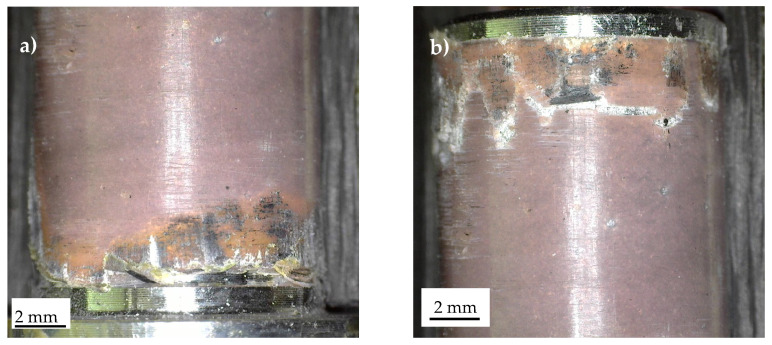
Characteristic fracture surface of a Series 1 specimen failed under variable-amplitude fatigue test. Detail of stress singularity point (**a**) 1 and (**b**) 2, according to Figure 3 (N_f_ = 6,593,200 cycles, D_M_ = 6.35).

**Figure 21:**
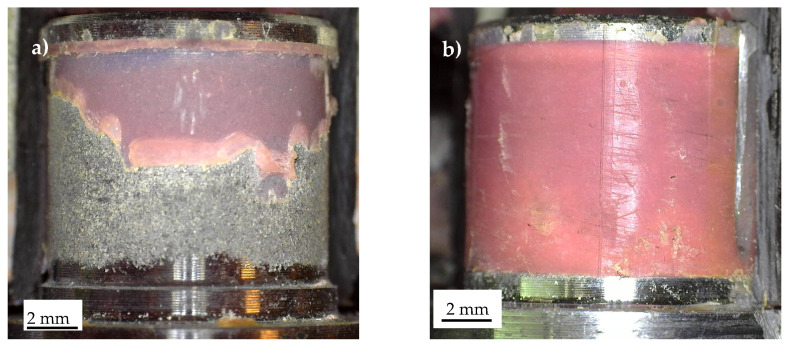
Characteristic fracture surfaces of a Series 2 specimen failed under variable-amplitude fatigue test (**a**) N_f_ = 720,800 cycles, D_M_ = 2.42 and (**b**) N_f_ = 212,000 cycles, D_M_ = 0.71.

Fracture surfaces obtained from variable-amplitude fatigue tests are reported in Figure 20 and Figure 21 for Series 1, 2, respectively. In Series 1, the precise site of crack initiation could not be identified, as illustrated in Figure 20a,b, which highlight points 1 and 2, respectively, where the singularity exponent s is the same. Nevertheless, Figure 20 indicates that the crack propagated along the composite–adhesive interface, consistent with the H_0_ values listed in Table 3.

Considering Series 2, Figure 21 presents fracture surfaces from two specimens: one that failed after N_f_ = 720,800 cycles (corresponding to D_M_ = 2.42) (Figure 21a) and another that failed after N_f_ = 212,000 cycles (corresponding to D_M_ = 0.71) (Figure 21b). A comparison of these two figures highlights distinct fatigue crack paths. Specifically, Figure 21a exhibits a failure mode more consistent with that observed under constant-amplitude fatigue loading (see Figure 18), whereas Figure 21b shows fatigue crack propagation exclusively along the adhesive–composite interface. Differently, Figure 21a depicts a more complex scenario. While Figure 21b reveals that the crack propagated at the adhesive–composite interface, Figure 21a shows a mixed failure mode involving both adhesive–composite and aluminium–adhesive crack propagation.

The analysis of the fracture surfaces revealed the following:

- Under quasi-static loading, all bonded joints exhibited a predominantly adhesive failure mode, occurring either at the composite–adhesive or aluminium–adhesive interfaces, depending on the joint configuration. For Series 4, a mixed failure mode was observed, combining adhesive failure at both interfaces with localized delamination through the unidirectional (UD) plies of the carbon tube.
- Under constant- and variable-amplitude fatigue tests, all Series 1 specimens showed crack propagation along the composite–adhesive interface (i.e., adhesive failure). In Series 2, crack propagation occurred along the composite–adhesive interface in four of nine specimens, while in the remaining cases, it took place along the aluminium–adhesive interface. For Series 4, crack propagation occurred along the composite–adhesive interface in five out of five specimens, with the remaining specimen failing at the aluminium–adhesive interface.
- Based on the fracture surface analyses, it was not possible to determine which of the four stress singularity points originated the fatigue crack initiation in constant- as well as variable-amplitude fatigue tests.

## 8. Conclusions

In this study, the static and fatigue behaviour of adhesively bonded carbon fibre–aluminium tubular joints were investigated, with particular emphasis on their application in lightweight suspension systems of a Formula SAE racing car subjected to constant- and variable-amplitude loading. The adopted methodology combined experimental testing, finite element simulations, and V-notch mechanics-based stress analysis to provide a comprehensive characterisation of the fatigue behaviour of the joints.

The main outcomes can be summarised as follows:

- Quasi-static tests demonstrated that nominal critical shear stress exhibited reduced scatter compared to the Generalised Stress Intensity Factor-based criterion H_0_, indicating its practicality for conservative design estimations. However, the H_0_ parameter proved more suitable for fatigue-related assessments, where local stress concentrations are expected to dominate failure initiation.
- While H_0_ is usually employed to correlate the fraction of the fatigue life associated with crack initiation—its natural domain of applicability—we have intentionally applied it here to the assessment of the total life, due to the lack of optical access to the nucleation region. Under constant-amplitude loading, the total fatigue life of the tested tubular joints was effectively correlated with the Generalised Stress Intensity Factor, H_0_, derived from linear elastic finite element models.
- The Miner linear cumulative damage model was applied to variable-amplitude fatigue conditions, representative of actual racing track load histories. The estimations obtained from Miner’s rule were predominantly on the safe side (Miner’s index ranging from 2.42 to 6.35), with only one case exhibiting a non-conservative estimate (Miner’s index of 0.71). Nevertheless, the intrinsic limitations of the model—such as neglecting load sequence effects—suggest adopting more advanced nonlinear damage accumulation approaches in future research.
- The findings support the use of singular stress field parameters, particularly H_0_, as a robust design and assessment tool for adhesively bonded tubular joints in applications where fatigue performance is critical. This approach enables a more localised and physically based characterisation of joint integrity compared to traditional nominal stress analyses.
- Post-mortem analysis of the failed specimens did not allow for an unambiguous determination of the crack initiation site; nevertheless, it provided valuable insights into the crack propagation path, which was found to be influenced by the specimen geometry. For Series 1 and Series 4 specimens, propagation predominantly occurred at the composite–adhesive interface, whereas in Series 2 specimens, it was generally observed at the aluminium–adhesive interface.

## Figures and Tables

**Table 1:** Layups and geometry details of tested specimens.

Series	Layup	Ø_1_ [mm]	Ø_2_ [mm]	L [mm]	h [mm]
1	[1 × W, 3 × UD, 1 × W, 3 × UD, 1 × W]	12	15	18	0.20
2	[1 × W, 1 × UD, 1 × W, 1 × UD, 1 × W]	12	15	9	0.40
4	[1 × W, 4 × UD, 1 × W]	12	15	18	0.20

**Table 2:** Elastic properties of UD and W lamina, adhesive, and aluminium insert.

	E_1_ [MPa]	E_2_ [MPa]	E_3_ [MPa]	G_12_ [MPa]	G_13_ [MPa]	G_23_ [MPa]	ν_12_	ν_13_	ν_23_
UD lamina (orthotropic)	219,200	6990	6990	4270	500	500	0.28	0.28	0.06
W lamina (orthotropic)	56,700	56,700	6990	3760	500	500	0.06	0.056	0.06
Epoxy adhesive (isotropic)	2870	2870	2870	1050	1050	1050	0.33	0.33	0.33
Aluminium insert (isotropic)	70,000	70,000	700,00	26,320	26,320	26,320	0.33	0.33	0.33

**Table 3:** Stress singularity exponent and Generalised Stress Intensity Factor H_0_ relevant to all test series.

Test Series	Stress Singularity Point	s	H_0_ [MPa·mm^−s^] *
1	1	−0.709	0.415
	2	−0.709	0.805
	3	−0.362	0.037
	4	−0.362	0.213
2	1	−0.709	0.944
	2	−0.709	1.766
	3	−0.362	0.104
	4	−0.362	0.382
4	1	−0.709	0.382
	2	−0.709	0.919
	3	−0.362	0.053
	4	−0.362	0.243

* Applied force F = 1000 N.

**Table 4:** Results of the quasi-static tensile tests.

Specimen	F_c_ [kN]	$\tau_{nom,c}$ [MPa]	H_0,c_ [MPa mm^0.709^]
1_1	14.77	21.76	11.89
1_2	14.58	21.48	11.74
2_1	8.44	24.87	14.9
2_2	8.87	26.14	15.67
4_1	19.01	28.01	17.46
4_2 *	4.26 *	6.27 *	3.91 *
4_4	12.67	18.67	11.64
Average value	13.06	23.49	13.88
Standard deviation	3.99	3.45	2.47
Coefficient of variation	0.31	0.15	0.18

* Not considered for statistical analysis.

**Table 5:** Summary of the applied variable-amplitude fatigue blocks.

Number of Cycles for Each Block Load	Peak Force [N]	Normalised Peak Force
500	5002.8	1.000
500	3458.6	0.691
500	2762	0.552
500	2171.1	0.434
1000	1815.9	0.363
1500	1448.7	0.290
2500	1203.2	0.241
3000	986.54	0.197
5000	806.76	0.161
6200	600.04	0.120

## Data Availability

The original contributions presented in this study are included in the article. Further inquiries can be directed to the corresponding author.
